# Photo/thermo-sensitive chitosan and gelatin-based interpenetrating polymer network for mimicking muscle tissue extracellular matrix

**DOI:** 10.1016/j.heliyon.2024.e39820

**Published:** 2024-10-24

**Authors:** Antonella Stanzione, Alessandro Polini, Francesca Scalera, Giuseppe Gigli, Lorenzo Moroni, Francesca Gervaso

**Affiliations:** a Università Del Salento, Dipartimento di Matematica e Fisica E. de Giorgi, Campus Ecotekne, via Monteroni, Lecce, 73100, Italy; b CNR NANOTEC – Institute of Nanotechnology, Campus Ecotekne, via Monteroni, Lecce, 73100, Italy; c Maastricht University, MERLN Institute for Technology-Inspired Regenerative Medicine, Complex Tissue Regeneration Department, Universiteitssingel 40, 6229ER, Maastricht, the Netherlands; d Università Del Salento, Dipartimento Medicina Sperimentale, Campus Ecotekne, via Monteroni, Lecce, 73100, Italy

## Abstract

The dynamic interplay between extracellular matrix (ECM), its 3D architecture and resident cells plays a pivotal role in cell behavior influencing essential processes like proliferation, migration, and differentiation. Matrix-based 3D culture systems have emerged as valuable tools to model organ and tissue interactions *in vitro*. A 3D matrix analog must possess high biocompatibility and fully reproduce the characteristics of the native tissue in terms of mechanical properties. In this regard, interpenetrating polymer networks (IPNs) are particularly attractive because of the high tunability of their physicochemical properties. In this study, a chitosan (Ch) and modified gelatin (GelMA) IPN with a sol-gel transition triggered by two external physical stimuli, UV light and temperature, was designed to mimic the muscle tissue ECM in terms of mechanical stiffness. The system was deeply characterized demonstrating to support not only the growth and viability of muscle cells embedded within the hydrogel, but also cell differentiation toward muscle phenotype.

## Highlights

• Chitosan and methacrylated gelatin were used to create a dual-sensitive hydrogel.
• The IPN resulted injectable, stable and sensitive to both light and temperature.
• The IPN mechanical properties resemble those of muscle tissues.
• The IPN supported C2C12 differentiation toward a muscle phenotype.
• The system represents a suitable 3D model for investigating neuromuscular diseases.

## Graphical abstract

**Figure:**
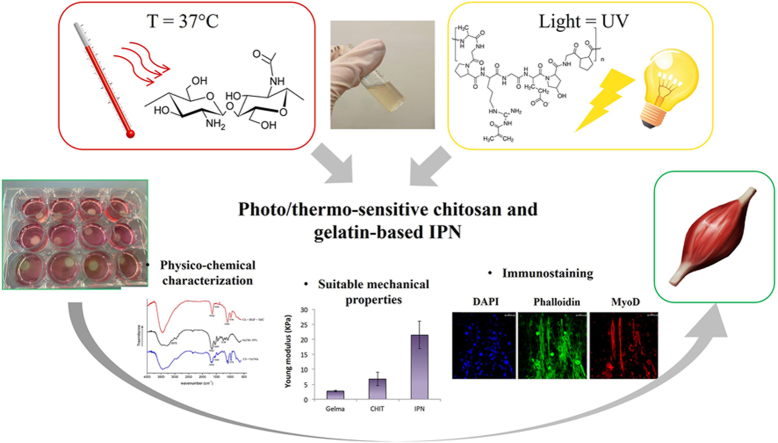

## 1 Introduction

The use of two-dimensional (2D) culture systems in research dates back to 1900, allowing for significant advancements in understanding physiological and pathological mechanisms witnessed *in vivo* [1]. They have provided valuable insights for understanding which mechanisms arise in certain pathological processes, studying the kinetics and targeting of new drugs, and evaluating cell metabolism [2]. However, despite their importance, 2D cell cultures present many limitations, such as limited morphology, lack of three-dimensional cell interaction, unsuitable cell differentiation and lack of flow and drainage of nutrients and wastes [3,4]. Among the above-highlighted drawbacks, a critical aspect is certainly represented by the lack of the three-dimensionality (3D) that all biological tissues present *in vivo*. Indeed, because of the absence of a 3D spatial organization, 2D *in vitro* models are not able to faithfully reproduce the native environment in which cells are immersed [5,6], that has been recognized fundamental for guiding cell fate and behavior [3]. The importance of the extracellular matrix (ECM) *in vivo* and ECM-cells crosstalk is nowadays evident and widely documented. ECM, in fact, plays a pivotal role on cell behavior in terms of adhesion [7], proliferation [8], migration [9], differentiation and cell death [10], providing cells with continuous peculiar stimuli and specific signals both in physiological and pathological processes [5,[11], [12], [13]]. In this regard, it has been observed that ECM continuously remodels and, in many complex pathologies this remodeling process is disrupted causing the ECM breakdown especially in terms of rigidity [12]. Indeed, the stiffness of the microenvironment in which cells live is a key factor influencing cell life and homeostasis [14,15]. Cells within a tissue are sensitive to mechanical stimuli arising from the surrounding environment and react by activating intracellular pathways related to mechanotrasduction [16,17]. Notably, increased ECM stiffness is associated with the translocation of the transcriptional regulators YAP (protein associated with Yes) and TAZ (PTZ binding motif) into the cell nucleus, leading to changes in chromatin levels and promoting cell proliferation, gene transcription and glycolysis [18,19]. Conversely, a reduction in matrix stiffness lowers glycolysis. Therefore, to reproduce a physiological environment or to study a pathological process, the stiffness of the environment surrounding the cells must be accurately reproduced [20,21]. Among the biomaterials commonly used in 3D culture systems, hydrogels exhibit significant similarities to the physiological ECM [22]. These hydrophilic polymeric networks not only mimic the mechanical and structural properties of the ECM, but also offer easy modulation of stiffness, allowing the creation of 3D microenvironments resembling various tissues [23,24]. Hydrogels stiffness can be modulated by tuning parameters such as gelling agent/crosslinker concentration, oxidation of the cysteines present in the side chains of the polymeric skeleton, or by introducing hydrophobic amino acids [25,26]. An innovative strategy to modulate hydrogel chemical-physical properties is represented by the so-called inter penetrating network polymers (IPN). Hydrogels used for 3D culture systems can be composed by one or more polymers, generating in the latter case either co-polymeric hydrogels or IPNs. An IPN is a polymeric system comprising two or more networks, which are at least partially interlaced on a molecular scale but not covalently bonded to each other and cannot be separated unless chemical bonds are broken. IPNs offer the great advantage, by combining the properties of the starting polymers, of generating a system with new, highly modulable properties, distinct from those of the original single polymers [[27], [28], [29]].

In this regard, 3D *in vitro* models of muscle tissue are fundamental to study muscle physiopathology in a highly representative environment and can help to deeper understand the cellular and molecular mechanisms involved in muscle contraction, formation and recovery, and the influence of drugs or toxins on muscle cells. Furthermore, 3D *in vitro* muscle models can be used to study specific muscle diseases, such as muscular dystrophy and other genetic and acquired pathologies, allowing to analyze the cellular and molecular alterations associated with these diseases and to develop new therapeutic strategies [30]. From a mechanical point of view, muscle tissue, can be defined as a complex system in which forces propagated both in axial and lateral direction [31]. Experimental analyses have revealed that the transverse Young's modulus of the striated muscle is 24.7 ± 3.5 kPa [32]. Furthermore, during the physiological processes of cell differentiation that are the basis of tissue homeostasis, muscle fibers undergo remodeling, resulting in increased stiffness [33,34]. To create an accurate hydrogel-based *in vitro* model of muscle tissue, it is therefore crucial to precisely tune the mechanical properties of the system to match the target tissue. In this regard, interpenetrating polymer networks (IPNs) offer an excellent solution. In the present work, we developed an *ad hoc* and innovative IPN hydrogel composed of chitosan and methacrylate gelatin (GelMA) that is dual sensitive, i.e., able to perform the transition from solution to gel through thermal (37 °C) and light (UV) stimulation. As it has been extensively reported in literature [[35], [36], [37], [38], [39], [40], [41]], chitosan solutions, in the presence of β-Glycerophosphate (βGP), remain liquid at room temperature, even at neutral pH, and are thermally sensitive, reaching the solid state (hydrogel) at body temperature. βGP plays three important roles in such a system: it *(i)* increases pH to the physiological range of 7.0–7.4, *(ii)* prevents the abrupt precipitation of chitosan fibers, and *(iii)* controls gel formation as temperature increases. The molecular mechanism of gelation includes several interactions between chitosan, βGP and water: 1) an increase in interchain hydrogen bonds as a consequence of the reduction of electrostatic repulsion due to the basic action of the salt, 2) a chitosan-βGP electrostatic attraction between the ammonium groups and the phosphate groups, respectively; 3) hydrophobic chitosan-chitosan interactions. Conversely, hydrogels based on methacrylamide-modified gelatin (GelMA), can form chemically stable materials through UV irradiation in the presence of water-soluble photoinitiators [41]. The photo-crosslinking results in the formation of a chemical network of modified gelatin. Here we explored the opportunity to prepare a mixed solution containing both gelatin, chitosan and their respective crosslinking promoters (i.e. salts and photoinitiator) and exploit two stimuli, UV and temperature) to create an IPN hydrogel stiffer than the two single counterparts. The proposed hydrogel system was extensively characterized to evaluate its ability to reproduce the mechanical properties of the muscle tissue and support the growth and differentiation of muscle cells embedded within it, towards a faithful 3D *in vitro* model of muscle tissue.

## 2 Materials and methods

### 2.1 Materials

Low molecular weight chitosan (degree of deacetylation 75–85 %, MW 50.000–190.000 Da, #448869), beta-glycerophosphate pentahydrate salt powder (βGP, MW 306.11 g mol^−1^, #35675), phosphate buffer (PB), sodium hydrogen carbonate (SHC, MW 84.007 g mol−1, #401676), hydrochloric acid (HCl), gelatin from bovine skin (type B, #9000-70-8), methacrylic anhydride (MW: 154.16 g/mol, # 276685), Irgacure 2959 photoinitiator (MW: 224.25, #410896), dextran molecules labelled with fluorescein isothiocyanate, i.e., 20k Da (#FD20) and 70 kDa (#FD70), primary anti-myosin, secondary antibody, goat anti-mouse IgG (H + L) conjugated with Alexa Fluor 555 and phalloidin–FITC were purchased from Sigma Aldrich (Milan, Italy). Spectro/Por molecular porous membrane tubes (MWCO 12–14.000,#08-801-244) were purchased from Fisher Scientific. Immortalized murine C2C12 muscle cells (#CRL-1772) were purchased from ATCC, mouse motor neuron-like hybrid cell line cells (NSC-34, #CLU140-A) from tedubio (Magenta, Italy). Dulbecco's Modified Eagle Medium (DMEM), glutamine, FBS, penicillin streptomycin were purchased from Corning (Amsterdam, Netherlands). Live and Dead ReadyProbes tests, Blue/Red assays were purchased from Thermo Fisher Scientific (Rodano, Italy).

### 2.2 Preparation of chitosan-based thermo-sensitive hydrogel

The Ch+βGP + SHC hydrogel was prepared following the protocol previously described [42]. The polymer solution was stored at 4 °C. For the preparation of the hydrogel, the gelling solution (#1 GA) was prepared starting from a 0.5M βGP solution obtained by dissolving βGP in Milli-Q water and a solution of SHC 0.125M obtained by dissolving SHC powder in Milli-Q water. The Ch+βGP + SHC hydrogel was prepared by cold mixing the Ch solution with the #1 GA solution with a ratio of 3:2 (v/v) [41]. The #1 GA solution was added drop by drop to the Ch solution and mixed manually with a spatula until a homogeneous solution was obtained. After mixing, the hydrogel was placed in a plastic mold at 37 °C for 2h in order to make the sol-gel transition.

### 2.3 Preparation of the gelatin-based photo-sensitive hydrogel (GelMA)

Before obtaining a hydrogel, gelatin was methacrylated in order to make the polymer photo-sensitive responsive in the presence of a photoinitiator, following a literature protocol described by Van Den Bulcke et al. [43]. 10 g of gelatin was added to 100 mL of PBS. Gelatin was kept under stirring at 60 °C until completely dissolved. Afterwards, 8 mL of methacrylic anhydride was added very slowly, dropwise, and the resulting emulsion was left under stirring at 60 °C for 3h. Next, 400 mL of preheated PBS were added to the emulsion and the resulting solution was kept at 40–50 °C for 15 min. The GelMA solution was then dialyzed against water for 8 days. At the end of the dialysis, the GelMA solution was transferred to a 50 mL falcon tube, frozen at −80 °C for at least 2 days, and lyophilized. The GelMA hydrogel was prepared by dissolving 50 mg (0.5 % w/v) of Irgacure 2959 in 10 mL of PBS at 80 °C and, once the photoinitiator was completely dissolved, 1 g of GelMA was added to obtain a 10 % (w/v) GelMA solution. The solution of GelMA and photoinitiator was placed inside a PDMS mold adherent to a microscope glass slide and irradiated with UV light for 4 min (wavelength = 365 nm, 0.18 W/cm^2^) to induce the photo-polymerization process.

### 2.4 Preparation of the Ch-GelMA photo/thermo-sensitive hydrogel

The photo/thermo-sensitive Ch-GelMA hydrogel was prepared by mixing a 3.33 % (wt/v) Ch polymer solution in 0.1M HCl with a #2 GA solution, composed of four components (namely GelMA, βGP, SHC and Irgacure 2959) dissolved in milliQ water, at a ratio of 3:2 (v/v) manually, by adding the #2 GA solution to the Ch solution until a slightly viscous and completely homogeneous solution was obtained. The Ch solution was prepared as previously described [41]. The #2 GA solution was prepared by dissolving the Irgacure 29959 powder at 1.25 % (w/v) in milliQ water, slowly increasing the temperature to 80 °C until complete dissolution. Once clear, 25 % (w/v) GelMA powder was added to the Irgacure 2959 solution, lowering the temperature to 40 °C to avoid modifications of the polymer. βGP and SHC powder were then added in order to obtain a solution at the final concentration of 0.75M and 0.375M for βGP and SHC, respectively. The #2 GA solution was stirred at r.t. until complete dissolution of the components.

### 2.5 Evaluation of pH and gelation test

The pH of solutions and hydrogels was measured before and after mixing by means of a pH-meter (SevenCompact S210, Mettler Toledo, Columbus, OH US) and pH indicator test strips, respectively. To evaluate the hydrogel gelation induced by the double stimulation, i.e., light and temperature, the ability of the prepared mix to slide along the walls of a vial was evaluated in the absence of stimulation and after light and temperature exposure (4 min of UV lamp and 10 min at 37 °C) [44].

### 2.6 Stability test

The stability test was carried out on n = 3 samples per type of hydrogel (Ch-GelMA, Ch+βGP + SHC and GelMA). Samples were weighed immediately after polymerization, time t = 0, and placed in PBS at 37 °C in an incubator. The samples were then weighed at certain timepoints (1, 2, 4, 24, 48 h and 7, 14, 21 days) in order to evaluate the degradation kinetics in a hydrolytic environment. The percentage weight loss, WL (%), over time was calculated applying equation (1):

(1) WL (%) = (W_0_ – W_i_)/(W_0_) ∗ 100

where *WL (%)* represents the weight loss as a percentage, *W*_*0*_ indicates the weight of the sample at t = 0, while *W*_*i*_ indicates the weight of the sample at the selected timepoints.

### 2.7 Swelling test

The swelling test was performed on n = 3 samples per type of hydrogel (Ch-GelMA, Ch+βGP + SHC and GelMA) by weighing the samples previously freeze-dried (dry state, t = 0) and then at pre-established timepoints after incubation in oven at 37 °C in PBS (5, 10, 15, 30, 60, 90 and 120 min) in order to evaluate the degree of swelling (SR%) applying equation (2):

(2) SR (%) = [(W_wet_ - W_dry_)/W_dry_] ∗ 100

where *SR (%)* represents the degree of swelling as a percentage, *W*_*dry*_ indicates the weight of the dry sample immediately after freeze-drying, *W*_*wet*_ indicates the weight of the samples after hydration at a certain timepoint.

### 2.8 Diffusion test

To guarantee the nutrient supply to the cells encapsulated within the hydrogels, their permeability to the passage of model molecules was evaluated by means of a diffusion assay. More in detail, two different dextran molecules labelled with fluorescein isothiocyanate, i.e., 20k Da (FD20) and 70 kDa (FD70), were used. For each type of hydrogel n = 3 samples were prepared. 500 μL of hydrogel were injected in a vial with a diameter of 15 mm, and hydrogels were polymerized according to their specific responsiveness. 1 mL of FD20 or FD70 solution, at a concentration of 1 mg/mL in PBS, was added above each disk of hydrogel in the vials [45]. The samples were incubated at 37 °C and, at different timepoints (0, 1, 6, 24 h), 200 μL of supernatant were collected and the absorbance at 490 nm was read using the Plate Reader Clario Star (BMG Labtech) in order to evaluate the residual concentration present in the supernatant, which represents an indirect indication of the dextran amount diffused in the hydrogel.

### 2.9 Rheological test

The rheological properties of Ch-GelMA, Ch+βGP + SHC and GelMA solutions, prior to impose the external stimuli, temperature and/or UV light irradiation, were assessed by using a rheometer (Physica MCR 301, Anton Paar). Shear rate sweeps at two different temperatures, 20 and 37 °C, were conducted by using a Peltier plate stage and a 25 mm parallel plate geometry. About 200 μL of each solution (in the case of GelMA, the solution was pre-heated up to 40 °C to be injectable) were deposited onto the stage, a gap distance of 300 μm was set and the samples were left to equilibrate at the set temperature for 10 min. Shear rate sweeps were performed in the range of 1–100 s^−1^. The test was performed in duplicate for each hydrogel solution.

### 2.10 Compression test

The stiffness of the three hydrogel formulations was investigated by means of compression tests carried out on the completely polymerized hydrogels and after their full hydration in PBS at r.t. (about 30 min, according to swelling test results). The test was performed using a universal testing machine (ZwickiLine 1 kN, Zwick Roell) with a 10N load cell in displacement control, with a displacement speed of 2 mm/min and up to 75 % deformation. Young's modulus was calculated for each sample as the slope of the initial linear part of the stress-strain curve (0–5%). The test was performed in triplicate for each hydrogel formulation.

### 2.11 Fourier transform IR spectroscopy (FTIR)

FTIR analysis was performed in order to evaluate the interactions that occur in the hydrogels between their components. The freeze-dried hydrogels of Ch+βGP + SHC, GelMA and their mix Ch-GelMA were crushed into powder and mixed with KBr (1:100, w/w). The IR spectral data were obtained using a FT/IR-6300 type A spectrophotometer (JASCO Inc) in the wavenumber range 4000–400 cm^−1^ at a resolution of 4 cm^−1^ with scan speed of 2 mm/s.

### 2.12 Scanning electron microscope (SEM) analysis

The porosity of the samples was studied by observing the samples by scanning electronic microscopy (SEM EVO 40, Carl Zeiss AG). Once polymerized, the hydrogels were frozen and lyophilized and then longitudinal and transversal sample sections were obtained. Before being observed, the samples were sputtered by means of a gold coating with a thickness of 10 nm (CCU-010 HV, Safematic GmbH). Images at different magnifications were acquired with an accelerating voltage of 5 kV and analyzed with ImageJ software (v. 1.8.0_172) for the evaluation of the pores. Briefly, 6 sample sections (3 of longitudinal plus 3 transversal ones, as representative of the entire macrostructure) for each hydrogel formulation were imaged and 25 pores were measured for each image. GraphPad Prism software (v. 8.4.2) was employed to perform statistical analysis, using one-way ANOVA analysis (the values were considered significant with p < 0.05).

### 2.13 Cell culture and cell encapsulation in hydrogels

All the hydrogels were tested with immortalized murine C2C12 muscle cells and mouse motor neuron-like hybrid cell line cells (NSC-34). Cells were cultured in Dulbecco's Modified Eagle Medium (DMEM) complemented with 2 mM glutamine, 10 % FBS, 100 U/mL penicillin and 100 g/mL streptomycin and incubated at 37 °C in the presence of 5 % CO_2_. For the encapsulation of C2C12 and NSC34 cells within the hydrogels, a cell density of 6x10^6^ and 2x10^6^ were chosen, respectively. Cells were encapsulated in the hydrogels in the sol phase and then polymerized by means of: *(i)* 10 min at 37 °C for the Ch+βGP + SHC hydrogels, *(ii)* 4 min of UV exposure for GelMA hydrogels, *(iii)* both 4 min of UV exposure and 10 min at 37 °C for Ch-GelMA hydrogels.

### 2.14 Live and dead assay

The viability of C2C12 and NSC34 cells in the hydrogels was evaluated using the Live and Dead ReadyProbes test, Blue/Red. The assay was performed on 100 μL of the three hydrogels, loaded with the two different cell types, at three different timepoints: 1, 3 and 7 days. The assay was performed by preparing a stock solution consisting of 2 drops of NucBlue live reagent and 2 drops of propidium iodide per mL of culture medium. The samples were then incubated for 1 h and protected from light in the incubator. At the end of the incubation the samples were washed in PBS and visualized by fluorescence microscopy (Axio Zoom.V16, Zeiss), exciting at 360 nm for the visualization of the NucBlue® Live reagent (live cells) and at 535 nm for propidium iodide (dead cells).

### 2.15 Differentiation of C2C12 cells

C2C12 cells were encapsulated in the three different hydrogel formulations and maintained in culture in proliferative medium for 4 days. On day 4, the medium was replaced with differentiation medium complemented with 2 % horse serum. Differentiation was induced for 7 days. Afterwards, cells were fixed and subjected to immunofluorescence.

### 2.16 Immunofluorescence staining

C2C12 cells differentiated in the hydrogels were analyzed by immunofluorescence microscopy. Samples were fixed in 4 % PFA in PBS for 20 min, permeabilized with Triton X-100 in PBS, incubated with 1 % BSA in PBS for 1 h and then with the primary anti-myosin in 1 % BSA in PBS (1:1000 v/v) overnight at 4 °C. Afterwards, the samples were incubated with a secondary antibody, goat anti-mouse IgG (H + L) conjugated with Alexa Fluor 555, in 1 % BSA in PBS (1:1000 v/v) for 1 h, stained with phalloidin–FITC in PBS (1:400 v/v) and incubated with DAPI solution in PBS (1:10000 v/v). The images were acquired by confocal microscopy (Zeiss LSM700).

## 3 Results

### 3.1 Gelation test and pH measurement

**Fig. 1:**
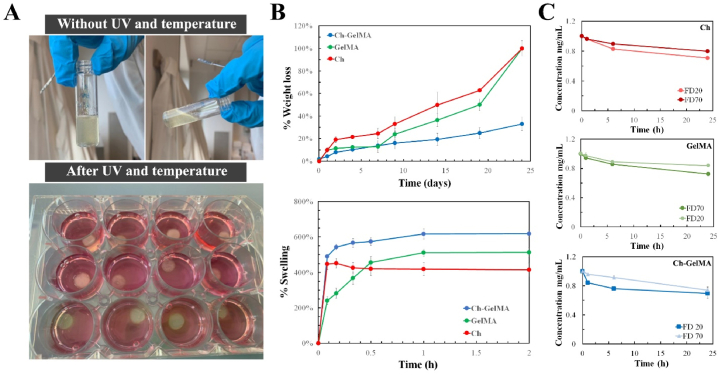
A) Visualization of the sol-gel transition: pictures of Ch-GelMA hydrogel before and after the double thermal and light stimulation. B) Evaluation of the degradability (top) and swelling (bottom) profiles of the three hydrogels. C) Evaluation of the permeability of the three hydrogel formulations (Ch, GelMA and Ch-GelMA) to the passage of molecules with different sizes (FD20 and FD70).

A hydrogel system based on chitosan and methacrylate gelatin was developed in order to obtain an IPN sensitive to both temperature and UV light. By preliminarily fine-tuning the ratio between the two polymers, Ch and GelMA, and the components of the gelling agent solution (#2 GA), it was possible to obtain a homogeneous and slightly viscous solution immediately after mixing (Fig. 1A). The solution, after being placed in a PDMS mold, was UV-exposed for 4 min and 10 min at 37 °C. The applied double stimulation led to a compact and homogeneous hydrogel. The thermo-sensitive Ch+βGP + SHC hydrogel showed to perform the sol-gel transition when placed at 37 °C (after mixing), as reported previously [42]. The GelMA-based hydrogel correctly carried out the sol-gel transition when exposed to UV light for 4 min. The pH of the polymeric solutions and of the gelling solutions was measured before and after mixing (Table 1). The solution resulting from mixing the components of the Ch-GelMA hydrogel displayed a physiological pH suitable for cell encapsulation.

**Table 1:** pH values and concentration (wt/v) of gelling agent, chitosan, GelMA and Ch-GelMA hydrogel solutions.

Samples	Initial concentration	Initial pH	Final concentration in hydrogel	Final pH
**GelMA solution**	25 %	8.6	10 %	–
**βGP solution**	0.75 M	8.6	0.2 M	–
**SHC solution**	0.375 M	8.5	0.05 M	–
**Ch solution**	3 %	5.5	2 %	–
**Ch-GelMA hydrogel**	–	–	–	7

### 3.2 Stability test

The stability test allowed to evaluate the degradation profile of the hydrogel in a hydrolytic environment by measuring the weight loss over time (Fig. 1B). All the hydrogel formulations showed high stability up to 7 days, with a weight loss percentage between 10 %, for Ch-GelMA and GelMA, and 20 %, for Ch+βGP + SHC. After 7 days the stability behavior of hydrogels started to differ: the WL (%) of Ch + BGP + SHC and GelMA samples showed an almost constant linear increase up to day 20, while the Ch-GelMA samples showed a superior stability over time (integrity up to 25 days). In particular, the results showed a weight loss of about 33 ± 6 % at day 24 for the Ch-GelMA hydrogels, significantly lower than GelMA and Ch+βGP + SHC samples that showed a weight loss of 50 ± 7 % and 40 ± 6.26 % at day 19 for the two hydrogels, respectively.

### 3.3 Swelling test

The swelling test was performed by monitoring the weight increase of the samples in PBS. All types of hydrogels showed a similar swelling behavior typical of hydrogels, reaching a maximum swelling value in the first hour of monitoring (Fig. 1B). In particular, after 1 h, Ch-GelMA samples showed a maximum weight gain value of 617 ± 22 %, while GelMA and Ch + BGP + SHC samples of 512 ± 27 % and 598 ± 16 %, respectively.

### 3.4 Diffusion test

The evaluation of the diffusion of nutrients across the hydrogels, fundamental for cell survival, was studied using dextran labelled with FITC at different molecular weights (20 kDa and 70 kDa). The results reported in Fig. 1B show that, at 24h of incubation, the Ch-GelMA hydrogels allowed the passage of 84 % of FD20 and 51 % of FD70 molecules, while the GelMA and Ch+βGP + SHC samples allowed the diffusion of 72 % and 68 % of FD20, and 41 % and 30 % of FD70, respectively.

### 3.5 Rheological test

**Fig. 2:**
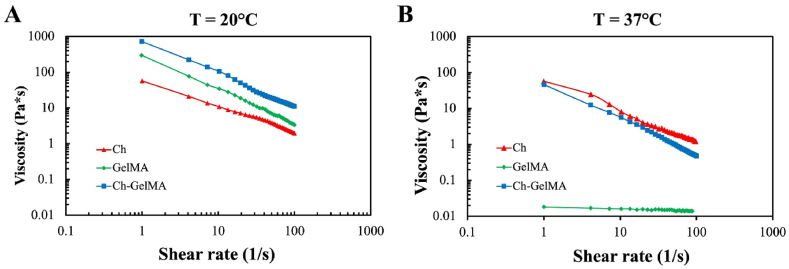
Rheological properties of Ch-GelMA, Ch+βGP + SHC and GelMA solutions prior to temperature and UV light exposure. A) Shear strain sweep tests performed at 20 °C. B) Shear strain sweep tests performed at 37 °C.

The rheological properties of the hydrogel solutions were investigated by shear rate sweeps at two different temperature, 20 and 37 °C, in the range 1–100 s^−1^. At 20 °C all the solutions resulted quite viscous and showed a shear-thinning behavior, characterized by a decrease in the shear stress as the shear rate increases (Fig. 2A). Ch-GelMA solution at 20 °C presented the highest viscosity with respect to the other solutions, according to the temperature-sensitive behavior of gelatin, i.e., liquid at temperature higher than 30 °C and solid at temperature lower than 30 °C, and also because of the higher total polymer content, i.e., chitosan plus GelMA. The less viscous solution was, as expected, the Ch+βGP + SHC, that is temperature sensitive in an opposite way with respect to gelatin polymer. At 37 °C, the GelMA solution viscosity drastically decreased, while the other hydrogel solutions maintained a shear-thinning behavior with a decrease in the viscosity values for the Ch-GelMA, due to the temperature-sensitive nature of the GelMA component (Fig. 2B). Overall, the rheological results confirmed that GelMA solution is strongly temperature sensitive, being very viscous at r.t. and becoming almost Newtonian as the temperature increases to 37 °C. The novel IPN here proposed, instead, showed an interesting shear-thinning behavior also at 37 °C despite the high GelMA content, because of the presence of chitosan and salt solutions that start the sol-gel transition at 37 °C.

### 3.6 Compression test

**Fig. 3:**
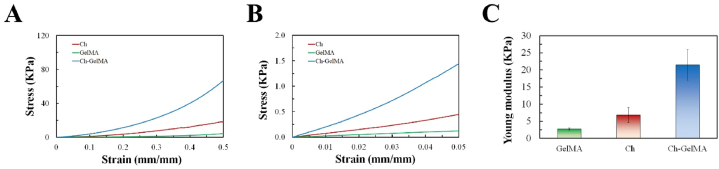
A, B) Compressive stress-strain curves of the three hydrogel formulations (Ch-GelMA, Ch+βGP + SHC and GelMA). C) Young modulus calculated as the slope of the stress-strain curve in the initial linear part (0–5%).

Compression test was carried out to determine the stiffness of the three hydrogel formulations. The results showed that for all the samples the stiffness increased as the strain increased, showing the typical compressive stress-strain behavior of hydrogel materials (Fig. 3A and B). The Young's modulus for Ch-GelMA, Ch+βGP + SHC and GelMA, resulted equal, respectively, to 21.44 ± 4.56, 6.82 ± 2.18 and 2.76 ± 0.27 Kpa (Fig. 3C). GelMA hydrogels showed a very low compressive stiffness according to literature data [46]. The novel Ch-GelMA photo/thermo-sensitive IPN showed a significantly higher stiffness than both GelMA and Ch+βGP + SHC, reaching the target value for muscle tissue (20–25 kPa).

### 3.7 Fourier transform IR spectroscopy (FTIR)

**Fig. 4:**
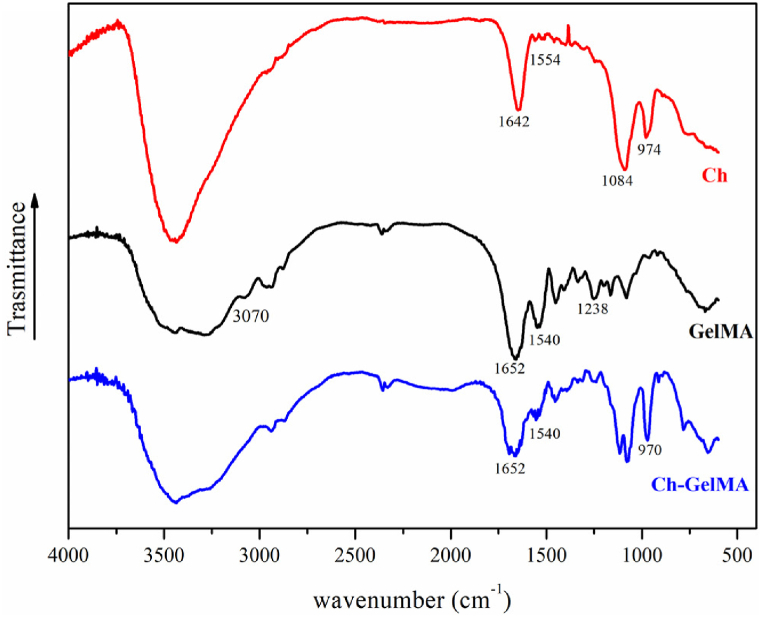
FTIR spectra of the comparison between hydrogels from single components (Ch, GelMA) and their mix (Ch-GelMA).

In Fig. 4, FTIR spectra of Ch+βGP + SHC, GelMA and Ch-GelMA formulations are reported. All spectra exhibited a strong and broad non-symmetric band in the range of wavenumber 3000-3700 cm^−1^ that results from overlapping of O-H and N-H stretching vibrations of functional groups engaged in hydrogen bonds. In the Ch+βGP + SHC spectrum, the characteristic absorption bands of chitosan were found [47]: C-H stretching at 2920–2878 cm^−1^, amide I (carbonyl, C-O) stretching at 1642 cm^−1^, deformation of the primary amino group (NH_2_) at 1554 cm^−1^ overlapping that of lower intensity of amide II (N-H) at 1556 cm^−1^. Moreover, the presence of the gelling agents, βGP and SHC, is shown respectively from the two intense bands for the phosphate groups at 1100-920 cm^−1^, and other two bands at 1468 and 1358 cm^−1^ for carbonate groups [48]. However, the bands corresponding to SHC had negligible intensity, perhaps due to the decomposition of SHC after mixing with the acidic chitosan solution [44]. FTIR spectrum of GelMA formulation showed the characteristic bands of amide I, amide II and amide III at 1652 cm^−1^, 1540 cm^−1^, and 1238 cm^−1^, respectively; the specific vibrations in the spectra at 3070 cm^−1^ confirmed the presence of peptide bonds (mainly N-H stretching) [46]. Ch-GelMA spectrum was very similar to pure GelMA due to the higher amount of GelMA in the final hydrogel with respect to the Ch content. Nevertheless, the specific chemical groups of both GelMA and Ch+βGP + SHC are observed and no additional characteristic absorption peaks or significant shifts in major peaks are identified, indicating the good blending of GelMA and Chitosan in the IPN system.

### 3.8 Scanning electron microscope (SEM) analysis

**Fig. 5:**
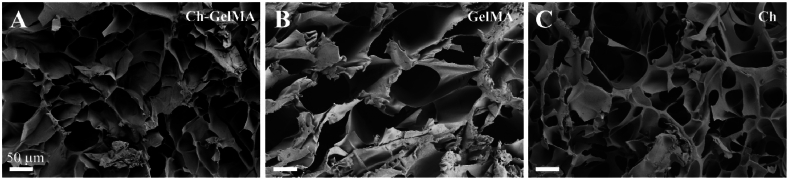
A-C) SEM images of longitudinal sections of the three hydrogel formulations (Ch-GelMA, GelMA and Ch).

SEM analysis of the longitudinal sections allowed to investigate the microstructure of the Ch-GelMA hydrogel after double crosslinking (UV + temperature). The SEM images (Fig. 5A–C) show an open porous structure of the hydrogel, very similar to the Ch+βGP + SHC and GelMA structure. The image analysis for Ch-GelMA revealed a pore size in the range 85.3 ± 33.2 μm, size slightly lower than the two hydrogels used as control, Ch+βGP + SHC and GelMA, that showed a pore size of 92.7 ± 21.2 μm and 101.7 ± 11.9 μm, respectively.

### 3.9 Live and dead assay

**Fig. 6:**
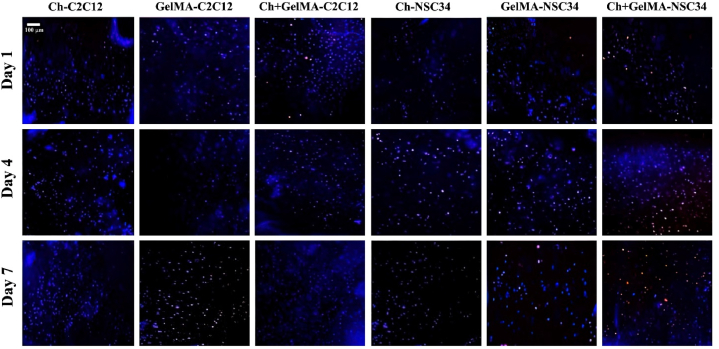
Live/Dead assay: viability analysis of C2C12 and NSC34 encapsulated cells in all the three hydrogel formulations up to day 7 of cell culture. Blue spots refer to living cells, red spots to dead cells. (For interpretation of the references to colour in this figure legend, the reader is referred to the Web version of this article.)

To evaluate the effects of hydrogel stiffness on cell viability, C2C12 cells were encapsulated in the photo/thermo-sensitive Ch-GelMA IPN hydrogel as well as in the GelMA and Ch+βGP + SHC hydrogels used as control (Fig. 6). As further control, also NSC34 cells, a neuron-like cell line [42], were encapsulated in the same materials to check the influence of the environment stiffness also on a cell line with a different phenotype. C2C12 cells showed excellent viability in the Ch-GelMA system, up to day 7, while NSC34 cells showed a high mortality, with the presence of many red spots (dead cells) on day 7, demonstrating to suffer being embedded in a microenvironment stiffer than their natural one. On the other hand, NSC34 cells showed optimal viability both in the thermo-sensitive Ch+βGP + SHC and photo-sensitive GelMA hydrogels, which have stiffness values in the range of nervous tissues. The viability values were also optimal up to day 7 for C2C12 cells in the Ch+βGP + SHC and GelMA samples.

### 3.10 Differentiation of C2C12 cells in hydrogel

**Fig. 7:**
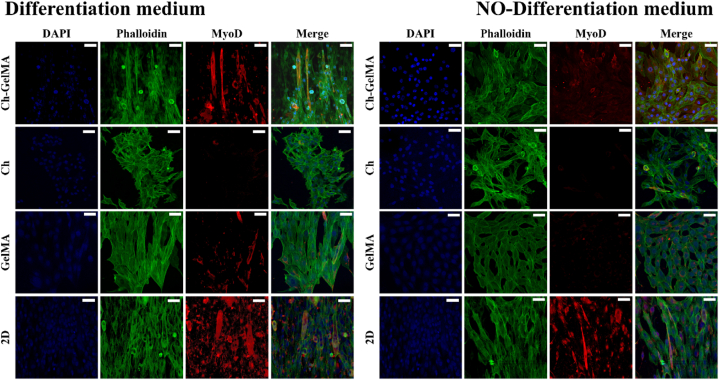
Fluorescent images of C2C12 cells encapsulated in all the types of hydrogels and cultured in either differentiation or non-differentiation media: nuclei, actin and MyoD are stained in blue, green and red, respectively. Cells were also cultured in 2D plastic plates as a control. Scale bars: 100 μm. (For interpretation of the references to colour in this figure legend, the reader is referred to the Web version of this article.)

C2C12 cells were encapsulated and induced to differentiate for 7 days. A parallel experiment was conducted, seeding cells in 2D culture plate as a control. Additionally, samples of cell-laden hydrogels were kept in proliferative medium, as further control. After seven days, the samples were immunostained and analyzed by confocal microscopy (Fig. 7). The cells encapsulated in Ch-GelMA IPN exhibited an elongated morphology and cellular fusion, typical of myocyte formation, confirming the strong ability of the IPN hydrogel to support cell viability and also demonstrating its suitability to mimic faithfully the microenvironment for their differentiation toward muscle tissue. On the other hand, cells encapsulated in the control formulations, i.e. Ch+βGP + SHC and GelMA hydrogels, despite an elongated morphology, did not show the formation of myocytes. Cells cultured in 2D culture plate, instead, showed appropriate morphology and cell fusion. Furthermore, cells encapsulated in different hydrogel formulations but not exposed to differentiation medium showed an elongated morphology but no cell fusion, confirming the importance of using differentiating medium for proper cell differentiation.

## 4 Discussion

*In vivo*, muscle tissue cells are continuously subjected to mechanical stimulation, essential for physiological function and cellular molecular mechanisms [31]. For this reason, in the development of 3D *in vitro* models that reproduce muscle tissues, it is vital to consider the mechanical properties of the target tissue to reproduce an environment suitable for cells, not only in terms of biocompatibility but also of functionality and performance [49]. Several IPN hydrogels have been developed in the last years for biomedical applications, such as tissue engineering, with the aim of increasing the stiffness of biopolymers-based hydrogels, that usually present excellent biological properties, but poor mechanical properties [[50], [51], [52]]. In particular, Suo and colleagues [53] developed semi-IPN and IPN GelMA-Chitosan hydrogels by the formation of covalent bonds and hydrophobic interactions through photo-crosslinking and basification. The prepared IPNs showed increased mechanical properties and high biocompatibility and were proposed as pre-formed scaffolds for subsequent cell seeding onto a lyophilized structure, while ours was designed for cell embedding, injection and culturing. These characteristics would be extremely useful in the case of developing more complex 3D *in vitro* models based on bioprinting and organ-on-chip technologies [54], or for *in vivo* (*in situ)* tissue engineering applications (e.g., to fill an open bone defect) [55].

With the aim of reproducing a 3D *in vitro* model for the culture and differentiation of muscle cells, in our study, an IPN hydrogel with mechanical properties similar to those of muscle tissue was designed and developed. The hydrogel was obtained by combining two polymers, chitosan and methacrylated gelatin, which allowed for the generation of a photo/thermo-sensitive hydrogel. Such hydrogel can undergo a sol-gel transition triggered by both *(i)* temperature stimulation, thanks to the presence of chitosan and βGP + SHC salts that induce at 37 °C physical crosslinking of chitosan due to electrostatic interactions, and *(ii)* light stimulation, due to the presence of gelatin, that was previously modified by introducing UV-sensitive methacrylic groups, and a photoinitiator. The obtained hydrogel showed an excellent sensitivity to the dual stimulation with the formation of a compact and uniform hydrogel, injectable before stimulation through a 22G needle and characterized by a physiological pH value, suitable for cell encapsulation [56]. Stability test showed that the developed protocol for the preparation of the photo/thermo-sensitive hydrogel led to the formation of a highly stable IPN in cell culture conditions over time, displaying minimal degradation rate and structural integrity beyond three weeks, crucial features for long-term 3D cultures involving cell differentiation and tissue maturation [57]. Swelling test revealed the IPN hydrogel exceptional ability to retain water, even higher than the single polymer hydrogels, GelMA and Ch + BGP + SHC, despite the superior durability in culture conditions. Diffusion tests, performed using FITC-labelled dextran molecules of different size [58], indicated the high permeability of the Ch-GelMA hydrogel to a broad range of molecules and nutrients involved in cell metabolism and nourishment, ensuring adequate supply to the encapsulated cells and further confirming its potential use in 3D culture systems.

The incorporation of chitosan and methacrylated gelatin, along with the specific gelling agents (βGP, SHC, and photoinitiator), resulted in the formation of a stable IPN hydrogel. This hydrogel exhibited robust mechanical properties, with a Young's modulus within the range typically observed in physiological muscle tissue (20–25 kPa) [32]. Indeed, the compression test demonstrated that the IPN system displayed unique mechanical properties that surpassed those of the individual components. This enhancement in compressive stiffness is likely due to the entanglement of the two cross-linked components, indicating the synergistic effect resulting from the IPN nature of the developed system [59].

The rheological analysis was conducted to investigate the material behavior at two different temperatures, 20 and 37 °C, in the range 1–100 s^−1^. The Ch-GelMA solution showed a higher viscosity at 20 °C compared to the Ch and GelMA solutions, attributed to a higher polymer content. GelMA showed a drastic decrease in viscosity at 37 °C, confirming its classic behavior [60]. In contrast, despite the high GelMA content in the IPN, the viscosity of the Ch-GelMA IPN did not undergo a sudden drop with temperature rise, confirming the effective interaction among the single materials and the saline component, responsible for the sensitivity to temperature and the correct sol-gel transition at 37 °C. FTIR analysis of IPN highlighted the interaction between the polymers and saline solutions. In particular, the presence of a high concentration of GelMA in the IPN hydrogel was highlighted by the characteristic peaks of amide I, amide II and amide III at 1652 cm^−1^, 1540 cm^−1^ and 1238 cm^−1^, respectively, overlapping the spectrum of GelMA formulation [46]. Despite the significant presence of GelMA, the peaks of the Ch+βGP + SHC system could still be detectable in the IPN spectrum. These included the peaks at 2920–2878 cm^−1^ attributable to chitosan and C-H bond, to the amide at 1642 cm^−1^, to deformation of the primary amino group (NH2) at 1554 cm^−1^. The peaks of the βGP and SHC salts were also observed, i.e. two intense bands for the phosphate groups at 1100-920 cm^−1^, and two other bands at 1468 and 1358 cm^−1^ for the carbonate groups [47]. In summary, FTIR spectral studies confirmed that the Ch+βGP + SHC thermo-sensitive responsive hydrogel is entrapped in UV-sensitive GelMA hydrogel only by the physical forces and not by any chemical interactions, in agreement with the IPN nature. Finally, SEM analysis highlighted the porous structure of the IPN, indicating its suitability for cellular encapsulation and facilitating the passage of nutrients, release of catabolites as well as the movement of the cells themselves [61].

The biological properties of the hydrogel samples were assessed using murine C2C12 muscle cells. The cells encapsulated in the hydrogel showed high viability up to 7 days of culture, as confirmed by the Live-dead assay that revealed minimal cell death. This test also demonstrated that the UV irradiation used to induce the crosslinking of the GelMA hydrogel component did not induce cell death [62]. The encapsulated cells were subsequently subjected to differentiation using a standard differentiation medium. C2C12 cells, stained by immunofluorescence and subjected to differentiation for five days, showed a change in their morphology from round to elongated, and fused muscle cell elements, characteristic of muscle differentiation, were observed [63].

Moreover, a neuron-like cell line, NSC34 cells [42], was also encapsulated in the same hydrogels, as a further control, to study the influence of the environment stiffness on cells with a different phenotype. *In vivo* and in physiological conditions, the two cell types experience highly different stiffness environments. C2C12 cells, being muscle cells, reside in an ECM characterized by higher stiffness, while NSC34 cells prefer a much softer environment. Compared to the controls, namely GelMA and Ch+βGP + SHC, Ch-GelMA, that displays a much higher stiffness, had a strong impact in terms of viability of the embedded cells, resulting in a markedly different behavior. C2C12 cells exhibited excellent viability in the Ch-GelMA system, whereas NSC34 cells experienced high mortality, suggesting that a microenvironment stiffer than their natural one can be cytotoxic [64]. On the other hand, their viability resulted optimal both in the thermo-sensitive Ch+βGP + SHC and photo-sensitive GelMA hydrogels, which possess stiffness values within the range of nervous tissues. To summarize, the differential responses of C2C12 cells and NSC34 cells in the hydrogels highlight the importance of matching the stiffness of the microenvironment to the specific cell type.

## 5 Conclusions

In this study, an *ad hoc* hydrogel was developed for culturing muscle cells in a 3D environment. In particular, an IPN with mechanical properties resembling those of muscle tissues was designed and deeply characterized. The IPN was obtained by combining two polymers, chitosan and methacrylated gelatin, which allowed for the generation of a photo/thermo-sensitive hydrogel. The system resulted injectable and sensitive to both light and temperature, stable in culture condition up to 24 days, exhibited excellent swelling and rheological properties, and supported the differentiation of C2C12 cells toward a muscle phenotype. In conclusion, the developed innovative photo/thermo-sensitive hydrogel based on chitosan and methacrylated gelatin can be considered an excellent system for the 3D culture of muscle cells and their differentiation. It represents a valuable tool for studying neuromuscular diseases and can be employed as a suitable 3D model for investigating related pathological mechanisms.

## CRediT authorship contribution statement

**Antonella Stanzione:** Writing – original draft, Methodology, Investigation, Data curation, Conceptualization. **Alessandro Polini:** Writing – review & editing, Supervision, Funding acquisition, Data curation, Conceptualization. **Francesca Scalera:** Investigation. **Giuseppe Gigli:** Supervision, Resources, Funding acquisition. **Lorenzo Moroni:** Supervision. **Francesca Gervaso:** Writing – review & editing, Supervision, Conceptualization.

## Declarations

Ethics statement: Commercially available cell lines were used in this study. In particular, immortalized murine C2C12 muscle cells (#CRL-1772) were purchased from ATCC, mouse motor neuron-like hybrid cell line cells (NSC-34, #CLU140-A) from tedubio (Magenta, Italy).

## Data availability

The raw/processed data required to reproduce these findings cannot be shared at this time as the data also forms part of an ongoing study.

## Declaration of competing interest

The authors declare the following financial interests/personal relationships which may be considered as potential competing interests: Alessandro Polini is Associate Editor (Materials Science Section) at Heliyon.
